# Appetitive Traits in a Population-Based Study of Polish Adolescents within the PLACE-19 Study: Validation of the Adult Eating Behavior Questionnaire

**DOI:** 10.3390/nu12123889

**Published:** 2020-12-19

**Authors:** Dominika Guzek, Dominika Skolmowska, Dominika Głąbska

**Affiliations:** 1 Department of Food Market and Consumer Research, Institute of Human Nutrition Sciences, Warsaw University of Life Sciences (WULS-SGGW), 159C Nowoursynowska Street, 02-776 Warsaw, Poland; 2 Department of Dietetics, Institute of Human Nutrition Sciences, Warsaw University of Life Sciences (WULS-SGGW), 159C Nowoursynowska Street, 02-776 Warsaw, Poland; dominika_skolmowska@sggw.edu.pl (D.S.); dominika_glabska@sggw.edu.pl (D.G.)

**Keywords:** appetitive traits, adolescents, secondary schools, Adult Eating Behavior Questionnaire (AEBQ), coronavirus-19, SARS-CoV-2, population-based study, PLACE-19 Study

## Abstract

Appetitive traits of food approach or food avoidance are commonly measured using the Adult Eating Behavior Questionnaire (AEBQ). However, there is no Polish version of the AEBQ validated for adolescents, and to the best of our knowledge, no study completed with the Polish version of the AEBQ has been published thus far. The present study aimed to validate the AEBQ in a population-based sample of Polish secondary school students and to assess differences in appetitive traits between boys and girls within the Polish Adolescents’ COVID-19 Experience (PLACE-19) Study. The PLACE-19 Study was conducted in a group of 2448 adolescents recruited in May 2020 through the random quota sampling of secondary schools. The AEBQ was used to assess food approach subscales (Food Responsiveness, Emotional Over-Eating, and Enjoyment of Food) and food avoidance subscales (Satiety Responsiveness, Emotional Under-Eating, Food Fussiness, and Slowness in Eating). To validate the questionnaire, the standardized factor loadings within confirmatory factor analysis (CFA) with weighted least squares (WLS) were analyzed, and invariance was verified. The CFA presented good model fit, with χ^2^ = 4826.105 (degrees of freedom (df) = 384), root mean square error of approximation (RMSEA) = 0.069 (90% confidence interval (CI): 0.067, 0.070), comparative fit index (CFI) = 0.90, and standardized root mean square residual (SRMR) = 0.08. The results revealed that, compared to the configural invariance model, the metric invariance model did not result in significantly decreased model fit, with ΔCFI = −0.002 and ΔRMSEA = −0.001, which were lower than the recommended cutoffs of 0.010 and 0.015, respectively. The scalar invariance model also did not result in significantly decreased fit of the model over the metric invariance model, with ΔCFI = −0.005 and ΔRMSEA = 0.000. Girls reported higher levels of Food Responsiveness (*p* < 0.0001), Emotional Over-Eating (*p* < 0.0001), Satiety Responsiveness (*p* < 0.0001), Emotional Under-Eating (*p* < 0.0001), and Slowness in Eating than boys (*p* < 0.0001), and the total AEBQ scores of girls were also higher (*p* < 0.0001). Positive inter-correlations were observed between all food approach subscales, as well as between Emotional Under-Eating and all food approach subscales for girls, boys, and the total sample; positive inter-correlations were also observed between the majority of food avoidance subscales. The present study confirmed the validity of the AEBQ in the studied population, and supported the associations between appetitive traits assessed using the AEBQ; it also indicated higher scores of both food approach and food avoidance subscales in girls than in boys in a population-based sample of Polish secondary school students.

## 1. Introduction

Dietary behaviors are developed rapidly from infancy to adolescence, and they are influenced not only by individual factors and household characteristics [1], but also by parent–child interactions and social interactions [2]. In the adolescence period, dietary behaviors may be crucial, as they contribute to further behaviors in adulthood and to the resultant health risks [3] associated with body mass [4] and dieting [5]. It is possible that appetitive traits may influence dietary behaviors [6]. Food approach traits are associated with eating onset dietary behaviors, whereas food avoidance traits are associated with food offset dietary behaviors [7]. Appetitive traits are defined as a set of persistent predispositions toward food [8,9] that interact with environmental factors and influence dietary behaviors and their consequences [6,8]. Appetitive traits are known to possess a strong genetic component [10], and they can be perceived as stable traits [11]. They are commonly measured in adults using the Adult Eating Behavior Questionnaire (AEBQ) developed by Hunot et al. [9], which has been newly validated in adolescents [12]. However, there is no Polish version of the AEBQ validated for adolescents, and to the best of our knowledge, no study completed with a Polish version of the AEBQ has been published thus far.

The lack of a reliable tool to measure appetitive traits in a Polish population of adolescents may be challenging, as such a tool may allow in-depth analysis of dietary behavior determinants. In the study conducted by Syrad et al. [7], associations between appetitive traits and young children’s eating patterns were studied using the Child Eating Behavior Questionnaire [13], from which the AEBQ was developed. The findings of this study emphasized that the behavioral expression of appetitive traits has been linked with the risk of obesity.

Appetitive traits have been associated with body mass index (BMI) in children and adults in numerous studies [6,9,14,15]. Food approach subscales are generally positively correlated with BMI, whereas food avoidance subscales are negatively correlated with BMI [6,9,14,15]. Because these traits can be measured across the life course and they have been shown to be continuous and stable traits [11], there is a need for longitudinal studies to examine the continuity and stability of appetitive traits across the lifespan [6].

Appetitive traits have been shown to be influenced by sex, as has been observed in both adults [16] and children [17]. This may be associated with the fact that females differ from males in appetitive traits that might make them more susceptible to eating in response to external food cues or less susceptible to feedback from internal satiety cues [18]. Such problems are especially important during adolescence, where females have been found to be more sensitive to internal satiety cues, tend to eat more slowly, and show higher levels of emotional over-eating than males [12]. Therefore, the possibility of assessing appetitive traits in adolescents may be an important public health goal, while also analyzing dietary behaviors.

Among the factors that may have influenced dietary behaviors and appetitive traits was the period of outbreak of coronavirus-19 disease (COVID-19) caused by severe acute respiratory syndrome coronavirus 2 (SARS-CoV-2), which affected all aspects of daily life [19]. A number of studies conducted during this period revealed changes in various dietary aspects, including overeating predispositions toward food. In the study conducted by Di Renzo et al. [20], the authors found that the consumption of homemade products and dishes increased, even for products that may have been purchased at the grocery store. Similarly, the study by Sidor and Rzymski [21] revealed that the majority of respondents reported eating more. Furthermore, in the study conducted by Phillipou et al. [22], it was observed that both restricting and binge eating behaviors were increased. Changes in eating and physical activity behavior were found to be influenced by the COVID-19 pandemic, as demonstrated by overeating behavior in a British adult population [23], while in an Australian adult population, 53.6% individuals reported overeating over the past two weeks [24]. Moreover, higher scores for food approach subscales and lower scores for food avoidance subscales may be associated with a tendency toward higher food frequency and higher food consumption, respectively, during eating occasions [10].

A number of studies conducted during the COVID-19 pandemic have revealed important determinants that might influence the dietary behaviors of adults [19,20,21], but thus far, no such studies in a population of adolescents have been published. Despite the fact that the determinants of dietary behaviors are generally numerous and complex [1,2], such dietary behaviors in adults during the COVID-19 pandemic can be explained by two major determinants: lockdown and stockpiling food at home [20]. Both determinants were also experienced by children and adolescents in this period, and may have also influenced their dietary behaviors.

Therefore, there is a need to validate the Polish version of the AEBQ for adolescents to measure appetitive traits in a population of adolescents during the COVID-19 pandemic. Thus, the present study aimed to validate the AEBQ in a population-based sample of Polish secondary school students and to assess differences in appetitive traits between boys and girls within the Polish Adolescents’ COVID-19 Experience (PLACE-19) Study.

## 2. Materials and Methods

### 2.1. Study Design

The PLACE-19 Study was designed as a population-based study to be conducted in a national sample of Polish secondary school students recruited from all regions and voivodeships of Poland. The PLACE-19 Study included a first phase (April 2020) that assessed hand hygiene [25] and personal protective behaviors [26] during the COVID-19 pandemic and a second phase (May 2020) that assessed food choice determinants [27] and appetitive traits during the COVID-19 pandemic. Secondary schools included in the first phase were excluded from the stratified sampling procedure in the second phase. The second phase was divided into two stages: the main stage took place from 29 April to 10 May 2020, and the subsidiary stage from 11 to 23 May 2020.

The PLACE-19 Study was conducted in accordance with the guidelines of the Declaration of Helsinki. All procedures received the approval of the Ethics Committee of the Institute of Human Nutrition Sciences of the Warsaw University of Life Sciences (SGGW-WULS) (No 20/2020). A written informed consent to participate in the study was obtained from all of the participants and from their parents/legal guardians.

### 2.2. Participants

The PLACE-19 Study was conducted in a group of secondary school students aged 15–20 years, who were chosen using a random quota sampling procedure, as described previously [27]. From the national secondary schools database, a representative sample of schools was chosen while using a stratified sampling procedure. As each region of Poland is divided into voivodeships, which are basic administrative units and include counties, the choice was conducted within voivodeships and counties.

During the main stage of the study, i.e., from 29 April to 10 May 2020, within each of 16 voivodeships, five counties were randomly chosen (80 counties), and within each of the 80 counties, 10 secondary schools were randomly chosen (800 secondary schools). After the main stage, it was verified whether the collected sample was representative of all regions of Poland, and on the basis of the result obtained, the subsidiary stage, from 11 to 23 May 2020, was conducted in only eight voivodeships (to correct the proportions of respondents from all regions of Poland). During the subsidiary stage of the study, within each of the eight chosen voivodeships, five counties were randomly chosen (40 counties), and within each of the 40 counties, 10 secondary schools were randomly chosen (400 secondary schools). The applied procedure resulted in 1200 secondary schools that were randomly chosen from all of the voivodeships of Poland to obtain a representative sample of secondary schools participating in the study.

### 2.3. Methods

The local boards of education participated in arrangements with secondary schools, if needed. During the organization, the principal from each secondary school was asked whether they agreed to their school participating in the study. After obtaining agreement from the principals and informed consent from the students and their parents/legal guardians to participate, the students received a link to the electronic questionnaire.

**Figure 1:**
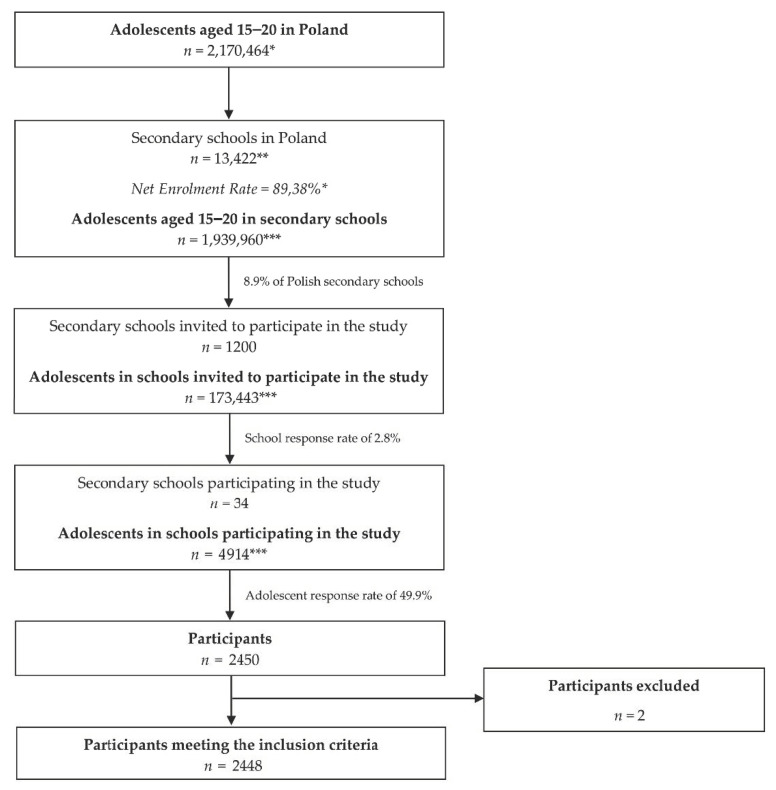
The flow chart of the school sampling and participants included. * by Statistics Poland [28,29]; ** by the Polish Ministry of National Education [30]; *** calculated on the basis of the data by Statistics Poland.

The completed questionnaires were anonymized, and no personal data were collected. While completing the questionnaire, the inclusion criteria were verified by asking about the secondary school (only students of randomly chosen schools were allowed to be included), age (only students aged 15–20 years were allowed to be included), and the informed consent to participate. After collecting the completed questionnaire, the forms were verified, and forms with any missing information or unreliable information were excluded. The information was considered to be unreliable if the respondent provided uniform answers across all of the questions, which was associated with failing to differentiate between answer choices (identical responses to all questions using the same scale). Finally, a total of 2448 participants were included in the analysis (Figure 1).

### 2.4. Measures

#### 2.4.1. Adult Eating Behavior Questionnaire (AEBQ)

Appetitive traits, including food approach and food avoidance traits, were assessed using the AEBQ questionnaire developed by Hunot et al. [9]. This questionnaire has been used in an adolescent population aged 11 to 18 years old [12] and younger adult populations aged 17 to 24 years old [31,32]. Moreover, the questionnaire has been validated to provide reliable data for adolescents [12].

The AEBQ was translated into Polish according to the recommendations by the World Health Organization (WHO) [33]. The questionnaire was first translated into Polish (forward translation) by a native Polish researcher who was familiar with the discipline, and subsequently, it was translated into English (backward translation) by an independent translator with no knowledge of the questionnaire. The results were then discussed by an expert panel of native Polish researchers who were fluent in English, and the questionnaire was corrected if needed.

The AEBQ is a 35-item self-report questionnaire that uses a five-point Likert scale. Items are clustered into eight subscales (three to five items each). Four food approach subscales assess: (1) Hunger (H)—five items; (2) Food Responsiveness (FR)—four items; (3) Emotional Over-Eating (EOE)—five items; and (4) Enjoyment of Food (EF)—three items. Food approach subscales define behaviors that involve movement and desire toward food [34]. Four food avoidance subscales assess: (1) Satiety Responsiveness (SR)—four items; (2) Emotional Under-Eating (EUE)—five items; (3) Food Fussiness (FF)—five items; and (4) Slowness in Eating (SE)—four items. Food avoidance subscales define behaviors that involve movement away from food [34]. The scores for each item are based on the Likert scale, and ratings are attributed to specific scores—from one point (“strongly disagree”) to five points (“strongly agree”), while some items are defined as reverse ones—from one point (“strongly agree”) to five points (“strongly disagree”). Based on the scores for each item, mean scores are calculated for each subscale.

As the confirmatory factor analysis (CFA) revealed that the seven-factor structure without the Hunger subscale was a better fit to the data than the original eight-factor model, further results are presented for the seven-factor structure. The same approach was reported in the study of Hunot-Alexander et al. [12], which was conducted to confirm the factor structure and reliability of the AEBQ in an adolescent sample. The detailed analyses for the original eight-factor model are presented in Supplementary Table S1.

#### 2.4.2. Sociodemographic Questionnaire

Participants were asked to answer additional questions on sex, age, and school attended (which allowed definition of the region of Poland and the type of school).

### 2.5. Statistical Analysis

The normality of the distribution of the data was verified using the Shapiro–Wilk test. Because of nonparametric distribution, the results were compared using the Mann–Whitney U test with Bonferroni correction, while relationships between subscales were analyzed using Spearman’s rank correlation coefficient.

The standardized factor loadings within the confirmatory factor analysis (CFA) with weighted least squares (WLS) were analyzed for internal reliability according to the common methodology applied for the AEBQ [12]. To assess the internal reliability of the data, McDonald’s ω was also used [35]. McDonald’s ω ≥ 0.7 was considered adequate [36]. CFA allows the imposition of a structure or model on the data, and tests how well that model “fits”, including testing (1) the number of factors, (2) whether the factors are correlated or uncorrelated, and (3) how items are associated with the factor [37]. To verify invariance, the analysis used was the same as that used in the Chinese validation of the AEBQ [14]. Tests of configural invariance, metric invariance, and scalar invariance were performed. A configural invariance test allows examination of whether the overall factor structure stipulated by measure fits well for the grouping variable in a sample. A metric invariance test examines whether the factor loadings are equivalent across the groups. A scalar invariance test is used to examine whether the item intercepts are equivalent across groups [38]. The following model fit indices were calculated: χ^2^, comparative fit index (CFI), root mean square error of approximation (RMSEA), and standardized root mean square residual (SRMR). The following cutoff criteria were applied: a value of 0.90 or more for CFI [39]; a value of 0.06 or less for RMSEA (good fit) [40]; a value of 0.08 or less for RMSEA (satisfactory fit) [41]; and a value of 0.08 or less for SRMR [39].

The external reliability of the Polish version of the AEBQ was verified through the test–retest analysis in a homogenic group of 20 young female respondents and measured twice over a two-week period. It refers to the reproducibility of scores on a measure over time in the same population [42]. The AEBQ was verified for its reproducibility by using the weighted κ statistic [43] and a cross-classification method, as the use of correlation coefficients to assess test–retest reliability is not recommended, because this method does not allow to detect systematic errors, and there are number of different methods used [42]. The weighted κ statistic with linear weighting and a cross-classification method was used to compare results within subscales, while respondents were divided into quartiles of their mean scores. In the cross-classification method, the results were interpreted as consistent if they were classified to the same or adjacent category.

Statistical significance was set at *p* ≤ 0.05. Statistical analysis was conducted using Statistica 13.3 (TIBCO Software Inc., Palo Alto, CA, USA) and JASP 0.14.0.0 (JASP Team 2020, University of Amsterdam, Amsterdam, The Netherlands).

## 3. Results

Descriptive characteristics of the study sample of Polish adolescents within the PLACE-19 Study are presented in Table 1.

The results of the test–retest reliability of the Polish version of the AEBQ are presented in Table 2. The analysis of weighted κ statistic values indicated fair to substantial agreement. According to the Landis and Koch [43] criteria, values ≤ 0 indicated no agreement, and values of 0–0.20, 0.21–0.40, 0.41–0.60, 0.61–0.80, and 0.81–1.0 indicated slight, fair, moderate, substantial, and almost perfect agreement, respectively. For almost all subscales, at least moderate agreement was observed, whereas for Satiety Responsiveness, fair agreement was noted.

The results of standardized factor loadings within the CFA with WLS obtained for the Polish version of the AEBQ are presented in Table 3. The results of standardized factor loadings within the CFA with WLS obtained for boys for the Polish version of the AEBQ are presented in Supplementary Table S2. The results of standardized factor loadings within the CFA with WLS obtained for girls for the Polish version of the AEBQ are presented in Supplementary Table S3.

Mean scores obtained for the AEBQ subscales and McDonald’s ω in this study of Polish adolescents are presented in Table 4. The scores obtained for items of the AEBQ subscales are presented in Supplementary Table S4. The analysis of McDonald’s ω values indicated fair to substantial agreement [36].

Sex invariance analyses of the AEBQ in the study of Polish adolescents are presented in Table 5. The CFA showed good model fit, with χ^2^ = 4826.105 (degrees of freedom (df) = 384), RMSEA = 0.069 (90% confidence interval (CI): 0.067, 0.070), CFI = 0.90, and SRMR = 0.08. The results revealed that, compared to the configural invariance model, the metric invariance model did not result in significantly decreased model fit, with ΔCFI = −0.002 and ΔRMSEA = −0.001, which were lower than the recommended cutoffs of 0.010 and 0.015, respectively. The scalar invariance model also did not result in significantly decreased fit of the model over the metric invariance model, with ΔCFI = −0.005 and ΔRMSEA = 0.000. This finding suggested a lack of response bias between boys and girls and allowed comparison of factor means across boys and girls.

Results from Table 6 show the comparison between mean AEBQ subscale scores for girls and boys in the study. The results revealed statistically significant differences, as girls reported higher levels of Food Responsiveness (*p* < 0.0001), Emotional Over-Eating (*p* < 0.0001), Satiety Responsiveness (*p* < 0.0001), Emotional Under-Eating (*p* < 0.0001), and Slowness in Eating than boys (*p* < 0.0001). Their total AEBQ scores were also higher than those for boys (*p* < 0.0001).

Correlations between AEBQ subscales in the sample of Polish adolescents are presented in Table 7. Positive inter-correlations were observed between all food approach subscales. Positive inter-correlations were also observed between the majority of food avoidance subscales, except for Food Fussiness and Emotional Under-Eating, as their correlation was negative, and except for Food Fussiness and Slowness in Eating, as their correlation was not statistically significant. Within the correlations between food approach subscales and food avoidance subscales, both positive and negative correlations were observed, while Emotional Under-Eating correlated with all food approach subscales. The total AEBQ score was also positively correlated with the scores for all of the subscales.

Correlations between AEBQ subscales in the sample of Polish boys are presented in Supplementary Table S5. Positive inter-correlations were observed between all food approach subscales. Positive inter-correlations were also observed between the majority of food avoidance subscales, except for Food Fussiness and Satiety Responsiveness, Food Fussiness and Emotional Under-Eating, and Food Fussiness and Slowness in Eating, as their correlations were not statistically significant. Within the correlations between food approach subscales and food avoidance subscales, both positive and negative correlations were observed, while Emotional Under-Eating was correlated with all food approach subscales. The total AEBQ score was positively correlated with the scores for all of the subscales.

Correlations between AEBQ subscales in the sample of Polish girls are presented in Supplementary Table S6. Positive inter-correlations were observed between all food approach subscales. Positive inter-correlations were also observed between the majority of food avoidance subscales, except for Food Fussiness and Emotional Under-Eating, as well as Food Fussiness and Slowness in Eating, as their correlations were negative, and except for Food Fussiness and Satiety Responsiveness, as their correlation was not statistically significant. Within the correlations between food approach subscales and food avoidance subscales, both positive and negative correlations were observed, while Emotional Under-Eating was correlated with all food approach subscales. The total AEBQ score was also positively correlated with the scores for all of the subscales.

## 4. Discussion

In the present study, a seven-factor, 30-item structured AEBQ without the Hunger items showed a satisfactory model fit. The results revealed that, compared to the configural invariance model, the metric invariance model did not result in significantly decreased model fit. The scalar invariance model also did not result in significantly decreased fit of the model over the metric invariance model. While comparing boys and girls, it was observed that girls reported higher levels of Food Responsiveness, Emotional Over-Eating, Satiety Responsiveness, Emotional Under-Eating, and Slowness in Eating than boys, while their total AEBQ scores were also higher. Positive inter-correlations were observed between all food approach subscales, as well as between Emotional Under-Eating and all food approach subscales for girls, boys, and the total sample; positive inter-correlations were also observed between the majority of food avoidance subscales.

The development study for the AEBQ [9], the study conducted by Hunot-Alexander et al. [12] for adolescents, and other studies, including the study of Mallan et al. [6], suggested that the Hunger subscale could be excluded from the AEBQ; hence, this approach was also chosen in the present study. Similarly, as reported in other previous studies [6,9,12], the original factor structure with the eight-factor model was compared with a seven-factor solution (excluding the Hunger subscale). The comparison revealed that the seven-factor model was suitable for the studied population of Polish adolescents. This finding corresponds with the results of Hunot-Alexander et al. [12], who showed that in their population of adolescents, the model without the Hunger subscale exhibited better results than those including this subscale. Thus, it can be stated that the reliability and validity of the AEBQ may be improved by removing the Hunger subscale.

Hunot-Alexander et al. [12] studied a sample of adolescents aged 11 to 18 years who were recruited from secondary schools in London; their results revealed similar associations between appetitive traits assessed by the AEBQ, as well as differences between girls and boys. In this study of English adolescents, the food approach subscales were positively inter-correlated, while Hunger was also positively correlated with Emotional Under-Eating [12]; a similar result was observed in the studied sample of Polish adolescents in the present study. The differences observed between girls and boys were also similar in both populations; in the study of English adolescents, girls showed higher scores for Emotional Over-Eating, Satiety Responsiveness, and Slowness in Eating [41], which was similar to that noted in the studied population of Polish adolescents. In the present study, girls showed higher scores for Food Responsiveness and Emotional Under-Eating, and similar to the study of Hunot-Alexander et al. [12], boys did not obtain higher scores for any of the subscales.

Hunot-Alexander et al. [12] also presented similar observations associated with correlations between appetitive traits assessed by the AEBQ, as well as differences between female and male respondents, and in this study, all of the food approach subscales were positively inter-correlated. In the study of Zickgraf and Rigby [16], no significant sex-dependent differences were observed for other subscales. However, it is not surprising that observations are inconsistent, as the abovementioned study of Zickgraf and Rigby [16] was conducted in a mixed sample of adolescents and adults, who were defined as patients pursuing bariatric surgery.

A comparison of the results of the AEBQ obtained for adolescents in the present study and in studies by other authors [12,16] with the results obtained for adults showed similarities in terms of correlations observed between appetitive traits [6,14,15], including Food Responsiveness and Satiety Responsiveness (correlation coefficient of 0.51), Satiety Responsiveness and Slowness in Eating (0.48) [6], Enjoyment of Food and Food Responsiveness (0.39) [14], Satiety Responsiveness and Slowness in Eating (0.38), and Emotional Under-Eating and Satiety Responsiveness (0.33) [15], and differences between scores obtained by female and male respondents [14,32]. Considering this aspect, it can be assumed that such observations may be typical for various populations.

In the present study, the AEBQ was administered during the unique period of the COVID-19 pandemic. It must be emphasized that the COVID-19 situation has a significant influence on mental health, as depression, anxiety, and stress are commonly observed psychological responses to the COVID-19 issue [44]. This also indicates that the COVID-19 pandemic may have a substantial effect on adolescents’ mental health, as a poll conducted by United Nations International Children’s Emergency Fund (UNICEF) showed that 27% of adolescents reported anxiety and 15% reported depression associated with the current COVID-19 issue [45].

Moreover, the COVID-19 pandemic may be associated with other problems related to the general interruption of routines and social interactions, which influence not only mental health [46], but also dietary behaviors and emotional eating [47,48]. Similarly, panic-buying or stockpiling of long-life foods, such as flour, sugar, dried pasta, rice, biscuits, and bottled and canned foods, may also contribute to increased consumption and overeating [24]. Last but not least, sedentary behaviors and a decrease in physical activity during the COVID-19 period should be considered, as they may also promote increased consumption resulting from food cravings [49].

The differences associated with sex and higher scores for female respondents than for male respondents for various AEBQ subscales may be explained by the general sex-dependent differences in food attitude. These observations indicate emotional eating, as confirmed by several studies, wherein the association between depression and emotional eating was found to be restricted to female respondents or was much stronger for female respondents than for male respondents [50]. Thus, it can be considered that in the present study, female respondents showed higher levels of Emotional Over- and Under-Eating than male respondents in response to stress associated with the epidemiologic situation. These results are consistent with general observations, which indicate that, especially for adolescents, there is an important association between perceived stress, worries, tension, anxiety, and resultant emotional eating, which is observed in girls but not in boys [51]. These observations are also noted in children, for example, in the International Study of Childhood Obesity, Lifestyle, and the Environment (ISCOLE) that studied a sample from the United Kingdom, girls showed a higher tendency for emotional eating than boys [52]. A comparison of sex-dependent dietary behaviors revealed that females have higher emotional susceptibility to disinhibition, but they also show a higher level of eating-related self-determined motivation than males [53]. Thus, women have a higher tendency toward overeating [54] and undereating [55], and these behaviors are transferred from parents to their daughters [56]. In an American study of Striegel-Moore et al. [57] conducted in a group of health organization members aged 18 to 35 years, a higher frequency of eating disorder symptoms was found in female patients than in male patients; furthermore, females diagnosed with binge eating disorders reported significantly higher body image dissatisfaction and drive for thinness than males [58]. This situation is explained by a cultural expectation of thinness in women [59], which results from internalized appearance standards [60] and causes their weight-related concerns [61]. It may also be associated with the influence of ovarian hormones [62] and menstrual cycle [63]; the mid-luteal phase increases emotional eating (as a result of ovarian hormone effects) [64], which may impact the associations for women.

In the analysis of results of the present study obtained using the AEBQ, it must be emphasized that this questionnaire minimizes participant burden when compared with similar questionnaires [16]. Moreover, the AEBQ measures eight appetitive traits, while other questionnaires, such as the Dutch Eating Behavior Questionnaire (DEBQ) [65] or the Three-Factor Eating Scale (TFEQ) [66], measure only two or three appetitive traits. In the present study, similar to other studies that analyzed the AEBQ in various populations [6,14,15], some significant associations were noted between the analyzed appetitive traits; this may result from the fact that the assessed appetitive traits are associated with each other, as they are influenced by individual characteristics and emotional responses. Macht [67] indicated that emotional overeating and emotional undereating are related to changes in eating behavior in response to emotional stress, including emotional control of food choice, emotional suppression of food intake, impairment of cognitive eating controls, eating to regulate emotions, and emotion-congruent modulation of eating.

Despite the fact that the present study is the first to use the AEBQ in a Polish population of adolescents and includes validation, some limitations of the study must be listed. The study was conducted during the COVID-19 pandemic, and the results may have been influenced by this specific period. Moreover, the study did not assess some potential confounders that may have influenced the obtained results, such as sociodemographic and lifestyle characteristics, household dietary practices, and health status, including diet-related diseases.

## 5. Conclusions

In a population of Polish adolescents, similarly to the studies by other authors, the seven-factor structure of the AEBQ without the Hunger subscale was a better model fit than that including the Hunger subscale, as it contributed to a higher reliability and validity. Girls reported higher levels of Food Responsiveness, Emotional Over-Eating, Satiety Responsiveness, Emotional Under-Eating, and Slowness in Eating than boys, while their total AEBQ scores were also higher. Positive inter-correlations were observed between all food approach subscales, as well as between Emotional Under-Eating and all food approach subscales for girls, boys, and the total sample; positive inter-correlations were also observed between the majority of food avoidance subscales.

The present study supports the associations between appetitive traits assessed while using the AEBQ; it also indicated higher scores of both food approach and food avoidance in girls than in boys in a population-based sample of Polish secondary school students.

## Figures and Tables

**Table 1:** Descriptive characteristics of the study sample of Polish adolescents within the PLACE-19 Study (*n* = 2448).

Characteristics		Results *n* (%)
Sex	Female	1552 (63.4%)
	Male	896 (36.6%)
Years	Mean ± SD	16.8 ± 1.1
	Median (range)	17 (15–20)
Region	North	270 (11.0%)
	North-West	160 (6.5%)
	South	479 (19.6%)
	South-West	335 (13.7%)
	Central	407 (16.6%)
	East	797 (32.6%)
Type of school	Comprehensive school	1238 (50.6%)
	Technical school	1210 (49.4%)

**Table 2:** Test–retest reliability of the Polish version of the Adult Eating Behavior Questionnaire (AEBQ) by Hunot et al. [9] (*n* = 2448).

AEBQ Subscale	Weighted κ Statistic	Cross-Classification—Share of Consistent Results (%)
Food Responsiveness	0.68	95
Emotional Over-Eating	0.60	95
Enjoyment of Food	0.52	90
Food avoidance subscales		
Satiety Responsiveness	0.36	80
Emotional Under-Eating	0.60	90
Food Fussiness	0.76	100
Slowness in Eating	0.68	100

**Table 3:** Standardized factor loadings within the confirmatory factor analysis (CFA) with weighted least squares (WLS) obtained for the Polish version of the Adult Eating Behavior Questionnaire (AEBQ) (*n* = 2448).

AEBQ Subscale	Items	Standardized Factor Loadings CFA with WLS	95% Confidence Interval	
			Lower	Upper
Food Responsiveness	I often feel hungry when I am with someone who is eating	0.529	0.490	0.567
	Given the choice, I would eat most of the time	0.574	0.540	0.609
	I am always thinking about food	0.412	0.382	0.441
	When I see or smell food that I like, it makes me want to eat	0.661	0.617	0.705
Emotional Over-Eating	I eat more when I’m annoyed	0.738	0.704	0.772
	I eat more when I’m worried	0.705	0.673	0.737
	I eat more when I’m upset	0.299	0.258	0.340
	I eat more when I’m anxious	0.625	0.595	0.655
	I eat more when I’m angry	0.517	0.489	0.546
Enjoyment of Food	I love food	0.881	0.845	0.917
	I enjoy eating	0.890	0.855	0.924
	I look forward to mealtimes	0.678	0.642	0.714
Satiety Responsiveness	I often leave food on my plate at the end of a meal	0.515	0.475	0.555
	I often get full before my meal is finished	0.671	0.629	0.713
	I cannot eat a meal if I have had a snack just before	0.446	0.407	0.486
	I get full easily	0.692	0.651	0.734
Emotional Under-Eating	I eat less when I’m worried	0.786	0.747	0.826
	I eat less when I’m angry	0.723	0.687	0.759
	I eat less when I’m upset	0.792	0.757	0.827
	I eat less when I’m annoyed	0.755	0.723	0.787
	I eat less when I’m anxious	0.818	0.784	0.852
Food Fussiness	I often decide that I don’t like a food before tasting it	0.727	0.696	0.757
	I refuse new foods at first	0.614	0.589	0.640
	I enjoy tasting new foods *	0.894	0.859	0.928
	I am interested in tasting new food I haven’t tasted before *	0.945	0.908	0.981
	I enjoy a wide variety of foods *	0.791	0.753	0.830
Slowness in Eating	I often finish my meals quickly *	0.626	0.597	0.655
	I eat more and more slowly during the course of a meal	0.867	0.829	0.906
	I eat slowly	0.628	0.593	0.663
	I am often last at finishing a meal	0.803	0.764	0.841

* Reverse items; CFA—confirmatory factor analysis; WLS—weighted least squares.

**Table 4:** Mean and median values and McDonald´s ω reliability scores for each AEBQ subscale in Polish adolescents (*n* = 2448).

AEBQ Subscale	Mean ± SD	Median (IQR)	McDonald’s ω
Food approach subscales			
Food Responsiveness	2.84 ± 0.66	2.75 (0.75)	0.66
Emotional Over-Eating	2.78 ± 0.55	2.60 (0.60)	0.79
Enjoyment of Food	3.58 ± 0.89	3.67 (1.33)	0.84
Food avoidance subscales			
Satiety Responsiveness	2.88 ± 0.70	2.75 (1.00)	0.70
Emotional Under-Eating	2.77 ± 0.83	2.60 (1.20)	0.88
Food Fussiness	2.65 ± 0.76	2.06 (1.20)	0.78
Slowness in Eating	2.92 ± 0.76	2.75 (1.00)	0.77
Total AEBQ	2.82 ± 0.34	2.80 (0.46)	0.75

**Table 5:** Sex invariance analyses of the Adult Eating Behavior Questionnaire (AEBQ) in Polish adolescents.

Model	χ^2^	df	CFI	RMSEA (90% CI)	SRMR	ΔCFI	ΔRMSEA
Boys	2032.779	384	0.847	0.069 (0.066, 0.072)	0.089	-	-
Girls	3307.669	384	0.845	0.070 (0.068, 0.072)	0.085	-	-
Configural invariance	5340.479 *	761	0.846	0.070 (0.068, 0.072)	0.086	-	-
Metric invariance	5402.345 *	784	0.844	0.069 (0.068, 0.071)	0.084	−0.002	−0.001
Scalar invariance	5557.986 *	814	0.840	0.069 (0.067, 0.071)	0.085	−0.004	0.000

df—Degrees of freedom; CFI—comparative fit index; RMSEA—root mean square error of approximation; CI—confidence interval; SRMR—standardized root mean square residual; ΔCFI—change in CFI relative to the preceding model; ΔRMSEA—change in RMSEA relative to the preceding model; * *p* < 0.01.

**Table 6:** The comparison between mean Adult Eating Behavior Questionnaire (AEBQ) subscale scores for Polish girls and boys (*n* = 2448).

AEBQ Subscale	Girls		Boys		*p*-Value *
	Mean ± SD	Median (IQR)	Mean ± SD	Median (IQR)	
Food approach subscales					
Food Responsiveness	2.89 ± 0.68	2.75 (0.75)	2.77 ± 0.61	2.75 (0.75)	0.0001
Emotional Over-Eating	2.84 ± 0.59	2.60 (0.60)	2.69 ± 0.46	2.60 (0.40)	0.0001
Enjoyment of Food	3.58 ± 0.90	3.67 (1.33)	3.57 ± 0.87	3.67 (1.33)	1.0000
Food avoidance subscales					
Satiety Responsiveness	2.99 ± 0.72	3.00 (1.00)	2.69 ± 0.61	2.50 (0.75)	0.0001
Emotional Under-Eating	2.93 ± 0.88	2.80 (1.60)	2.51 ± 0.66	2.20 (1.00)	0.0001
Food Fussiness	2.68 ± 0.78	2.60 (1.20)	2.62 ± 0.71	2.60 (1.20)	0.1015
Slowness in Eating	3.03 ± 0.77	3.00 (1.00)	2.74 ± 0.70	2.50 (1.00)	0.0001
Total AEBQ	2.89 ± 0.34	2.89 (0.46)	2.71 ± 0.30	2.69 (0.43)	0.0001

* Mann–Whitney U test with Bonferroni correction.

**Table 7:** Correlations between Adult Eating Behavior Questionnaire (AEBQ) subscales in Polish adolescents (*n* = 2448).

AEBQ Subscale	FR	EOE	EF	SR	EUE	FF	SE
Food Responsiveness (FR)	-						
Emotional Over-Eating (EOE)	0.23 **	-					
Enjoyment of Food (EF)	0.61 **	0.18 **	-				
Satiety Responsiveness (SR)	0.13 **	0.08 **	0.00	-			
Emotional Under-Eating (EUE)	0.28 **	0.07 **	0.12 **	0.41 **	-		
Food Fussiness (FF)	−0.20 **	−0.01	−0.31 **	0.05 *	−0.08 **	-	
Slowness in Eating (SE)	0.04	0.02	0.01	0.34 **	0.22 **	−0.04	-
Total AEBQ	0.58 **	0.59 **	0.41 **	0.57 **	0.65 **	0.17 **	0.45 **

* *p* ≤ 0.05; ** *p* ≤ 0.001.

## Supplementary Materials

The following are available online at https://www.mdpi.com/2072-6643/12/12/3889/s1, Table S1. Standardized factor loadings within the confirmatory factor analysis (CFA) with weighted least squares (WLS) for eight factors obtained for the Polish version of the Adult Eating Behavior Questionnaire. Table S2. Standardized factor loadings within the confirmatory factor analysis (CFA) with weighted least squares (WLS) obtained for boys for the Polish version of the Adult Eating Behavior Questionnaire (AEBQ). Table S3. Standardized factor loadings within the confirmatory factor analysis (CFA) with weighted least squares (WLS) obtained for girls for the Polish version of the Adult Eating Behavior Questionnaire (AEBQ). Table S4. The scores for items of the Adult Eating Behavior Questionnaire (AEBQ) in Polish adolescents. Table S5. Correlations between Adult Eating Behavior Questionnaire (AEBQ) subscales in Polish boys. Table S6. Correlations between Adult Eating Behavior Questionnaire (AEBQ) subscales in Polish girls.
