# Supplementary material for: Appetitive Traits in a Population-Based Study of Polish Adolescents within the PLACE-19 Study: Validation of the Adult Eating Behavior Questionnaire
Source: Nutrients. 2020 Dec 19;12(12):3889. doi: 10.3390/nu12123889 (PMC7766569; doi:10.3390/nu12123889)
Supplement: Supplementary file 1 [file nutrients-12-03889-s001.pdf]

nutrients

Article

Appetitive Traits in a Population-Based Study of Polish Adolescents: Validation of the Adult Eating Behaviour Questionnaire (AEBQ) and Assessment within PLACE-19 Study

Dominika Guzek [1]*, Dominika Skolmowska [2], Dominika Głąbska [2]

Table S1. Standardized factor loadings within Confirmatory Factor Analysis (CFA) with Weighted Least Squares (WLS) for 8-factors obtained for the Polish version of the Adult Eating Behavior Questionnaire.

AEBQ subscale	Items	Standardized factor loadings CFA with WLS	95% Confidence Interval	
			Lower	Upper
Hunger	I often notice my stomach rumbling	0.377	0.339	0.416
	If I miss a meal I get irritable	0.283	0.245	0.321
	I often feel so hungry that I have to eat something right away	0.463	0.427	0.499
	I often feel hungry	0.579	0.547	0.612
	If my meals are delayed I get light-headed	0.141	0.110	0.172
Food responsiveness	I often feel hungry when I am with someone who is eating	0.444	0.408	0.480
	Given the choice, I would eat most of the time	0.476	0.440	0.512
	I am always thinking about food	0.301	0.267	0.335
	When I see or smell food that I like, it makes me want to eat	0.361	0.327	0.395
Emotional over-eating	I eat more when I'm annoyed	0.577	0.545	0.608
	I eat more when I'm worried	0.529	0.497	0.561
	I eat more when I'm upset	0.406	0.370	0.443
	I eat more when I´m anxious	0.490	0.461	0.520
	I eat more when I'm angry	0.430	0.400	0.459
Enjoyment of food	I love food	0.652	0.625	0.678
	I enjoy eating	0.643	0.618	0.668
	I look forward to mealtimes	0.588	0.558	0.618
Satiety responsiveness	I often leave food on my plate at the end of a meal	0.508	0.475	0.541
	I often get full before my meal is finished	0.545	0.509	0.580
	I cannot eat a meal if I have had a snack just before	0.367	0.333	0.401
	I get full up easily	0.529	0.497	0.560
Emotional under-eating	I eat less when I'm worried	0.714	0.683	0.745
	I eat less when I'm angry	0.683	0.654	0.713
	I eat less when I'm upset	0.713	0.683	0.743

	I eat less when I'm annoyed	0.691	0.663	0.720
	I eat less when I'm anxious	0.728	0.700	0.756
Food fussiness	I often decide that I don't like a food, before tasting it	0.727	0.696	0.757
	I refuse new foods at first	0.614	0.589	0.640
	I enjoy tasting new foods*	0.777	0.754	0.801
	I am interested in tasting new food I haven't tasted before*	0.829	0.805	0.854
	I enjoy a wide variety of foods*	0.625	0.597	0.652
Slowness in eating	I often finish my meals quickly*	0.626	0.597	0.655
	I eat more and more slowly during the course of a meal	0.821	0.794	0.848
	I eat slowly	0.546	0.515	0.576
	I am often last at finishing a meal	0.755	0.729	0.781

* Reverse items; CFA – Confirmatory Factor Analysis; WLS – Weighted Least Squares.

Table S2. Standardized factor loadings within Confirmatory Factor Analysis (CFA) with Weighted Least Squares (WLS) obtained for boys for the Polish version of the Adult Eating Behavior Questionnaire (AEBQ).

AEBQ subscale	Items	Standardized factor loadings CFA with WLS	95% Confidence Interval	
			Lower	Upper
Food Responsiveness	I often feel hungry when I am with someone who is eating	0.538	0.472	0.603
	Given the choice, I would eat most of the time	0.480	0.427	0.532
	I am always thinking about food	0.454	0.414	0.494
	When I see or smell food that I like, it makes me want to eat	0.661	0.587	0.735
Emotional Over-Eating	I eat more when I'm annoyed	0.628	0.578	0.677
	I eat more when I'm worried	0.563	0.519	0.607
	I eat more when I'm upset	0.399	0.333	0.465
	I eat more when I´m anxious	0.565	0.522	0.608
	I eat more when I'm angry	0.488	0.447	0.528
Enjoyment of Food	I love food	0.863	0.805	0.920
	I enjoy eating	0.865	0.808	0.921
	I look forward to mealtimes	0.669	0.609	0.729
Satiety Responsiveness	I often leave food on my plate at the end of a meal	0.519	0.466	0.572
	I often get full before my meal is finished	0.561	0.493	0.629
	I cannot eat a meal if I have had a snack just before	0.380	0.321	0.438
	I get full up easily	0.626	0.560	0.693
Emotional Under-Eating	I eat less when I'm worried	0.645	0.593	0.696
	I eat less when I'm angry	0.558	0.509	0.608
	I eat less when I'm upset	0.639	0.594	0.685
	I eat less when I'm annoyed	0.575	0.533	0.618
	I eat less when I'm anxious	0.655	0.610	0.699
Food Fussiness	I often decide that I don't like a food, before tasting it	0.309	0.248	0.370
	I refuse new foods at first	0.271	0.213	0.328
	I enjoy tasting new foods*	0.869	0.813	0.925
	I am interested in tasting new food I haven't tasted before*	0.912	0.854	0.971
	I enjoy a wide variety of foods*	0.763	0.702	0.824
Slowness in Eating	I often finish my meals quickly*	0.439	0.381	0.496
	I eat more and more slowly during the course of a meal	0.778	0.721	0.836
	I eat slowly	0.589	0.535	0.643
	I am often last at finishing a meal	0.703	0.643	0.763

* Reverse items; CFA – Confirmatory Factor Analysis; WLS – Weighted Least Squares.

Table S3. Standardized factor loadings within Confirmatory Factor Analysis (CFA) with Weighted Least Squares (WLS) obtained for girls for the Polish version of the Adult Eating Behavior Questionnaire (AEBQ).

AEBQ subscale	Items	Standardized factor loadings CFA with WLS	95% Confidence Interval	
			Lower	Upper
Food Responsiveness	I often feel hungry when I am with someone who is eating	0.533	0.484	0.581
	Given the choice, I would eat most of the time	0.620	0.575	0.665
	I am always thinking about food	0.454	0.414	0.494
	When I see or smell food that I like, it makes me want to eat	0.656	0.602	0.711
Emotional Over-Eating	I eat more when I'm annoyed	0.779	0.734	0.825
	I eat more when I'm worried	0.762	0.718	0.806
	I eat more when I'm upset	0.403	0.337	0.469
	I eat more when I´m anxious	0.655	0.615	0.694
	I eat more when I'm angry	0.532	0.494	0.571
Enjoyment of Food	I love food	0.890	0.845	0.936
	I enjoy eating	0.905	0.861	0.949
	I look forward to mealtimes	0.684	0.639	0.729
Satiety Responsiveness	I often leave food on my plate at the end of a meal	0.519	0.466	0.572
	I often get full before my meal is finished	0.704	0.649	0.758
	I cannot eat a meal if I have had a snack just before	0.449	0.396	0.501
	I get full up easily	0.710	0.657	0.763
Emotional Under-Eating	I eat less when I'm worried	0.805	0.752	0.858
	I eat less when I'm angry	0.774	0.726	0.822
	I eat less when I'm upset	0.825	0.776	0.874
	I eat less when I'm annoyed	0.817	0.773	0.861
	I eat less when I'm anxious	0.862	0.815	0.908
Food Fussiness	I often decide that I don't like a food, before tasting it	0.309	0.248	0.370
	I refuse new foods at first	0.339	0.290	0.388
	I enjoy tasting new foods*	0.909	0.865	0.953
	I am interested in tasting new food I haven't tasted before*	0.963	0.916	0.998
	I enjoy a wide variety of foods*	0.807	0.758	0.856
Slowness in Eating	I often finish my meals quickly*	0.439	0.381	0.496
	I eat more and more slowly during the course of a meal	0.894	0.844	0.945
	I eat slowly	0.640	0.594	0.686
	I am often last at finishing a meal	0.827	0.778	0.876

* Reverse items; CFA – Confirmatory Factor Analysis; WLS – Weighted Least Squares.

Table S4. The scores for items of the Adult Eating Behavior Questionnaire (AEBQ) in Polish adolescents.

Subscale	Items	Mean	95% Confidence Interval
**Food approach subscales**			
Food Responsiveness	I often feel hungry when I am with someone who is eating	2.72	2.68–2.75
	Given the choice, I would eat most of the time	2.53	2.50–2.57
	I am always thinking about food	2.37	2.34–2.40
	When I see or smell food that I like, it makes me want to eat	3.75	3.71–3.79
Emotional Over-Eating	I eat more when I'm annoyed	2.62	2.58–2.66
	I eat more when I'm worried	2.52	2.48–2.55
	I eat more when I'm upset	3.86	3.82–3.89
	I eat more when I´m anxious	2.50	2.46–2.53
	I eat more when I'm angry	2.42	2.39–2.45
Enjoyment of Food	I love food	3.86	3.82–3.90
	I enjoy eating	3.78	3.74–3.82
	I look forward to mealtimes	3.09	3.05–3.13
**Food avoidance subscales**			
Satiety Responsiveness	I often leave food on my plate at the end of a meal	2.68	2.65–2.72
	I often get full before my meal is finished	3.01	2.97–3.05
	I cannot eat a meal if I have had a snack just before	2.66	2.63–2.70
	I get full up easily	3.16	3.12–3.20
Emotional Under-Eating	I eat less when I'm worried	2.93	2.89–2.98
	I eat less when I'm angry	2.76	2.72–2.80
	I eat less when I'm upset	2.79	2.75–2.83
	I eat less when I'm annoyed	2.66	2.63–2.70
	I eat less when I'm anxious	2.72	2.68–2.76
Food Fussiness	I often decide that I don't like a food, before tasting it	3.06	3.01–3.10
	I refuse new foods at first	2.69	2.65–2.72
	I enjoy tasting new foods*	2.53	2.49–2.57
	I am interested in tasting new food I haven't tasted before*	2.55	2.50–2.59
	I enjoy a wide variety of foods*	2.44	2.40–2.48
Slowness in Eating	I often finish my meals quickly*	3.03	2.99–3.07
	I eat more and more slowly during the course of a meal	2.71	2.68–2.75
	I eat slowly	3.08	3.04–3.12
	I am often last at finishing a meal	2.88	2.84–2.92

[*] Reverse items; * $p \leq 0.05$; ** $p \leq 0.001$.

Table S5. Correlation between Adult Eating Behavior Questionnaire (AEBQ) subscales in Polish boys.

AEBQ subscale	FR	EOE	EF	SR	EUE	FF	SE
Food Responsiveness (FR)	-						
Emotional Over-Eating (EOE)	0.18**	-					
Enjoyment of Food (EF)	0.58**	0.09*	-				
Satiety Responsiveness (SR)	0.19**	0.11*	0.04	-			
Emotional Under-Eating (EUE)	0.35**	0.25**	0.18**	0.31**	-		
Food Fussiness (FF)	-0.24**	0.01	-0.33**	0.05	-0.11	-	
Slowness in Eating (SE)	0.001	0.01	-0.02	0.29**	0.08*	0.04	-
Total AEBQ	0.60**	0.63**	0.46**	0.54**	0.63**	0.16**	0.39**

* $p \leq 0.05$; ** $p \leq 0.001$.

Table S6. Correlation between Adult Eating Behavior Questionnaire (AEBQ) subscales in Polish girls.

AEBQ subscale	FR	EOE	EF	SR	EUE	FF	SE
Food Responsiveness (FR)	-						
Emotional Over-Eating (EOE)	0.25**	-					
Enjoyment of Food (EF)	0.63**	0.23**	-				
Satiety Responsiveness (SR)	0.07*	0.03	-0.04	-			
Emotional Under-Eating (EUE)	0.22**	-0.06*	0.08*	0.41**	-		
Food Fussiness (FF)	-0.18**	-0.03	-0.30**	0.04	-0.08*	-	
Slowness in Eating (SE)	0.04	-0.01	0.02	0.32**	0.23**	-0.09**	-
Total AEBQ	0.57**	0.55**	0.40**	0.53**	0.62**	0.20**	0.45**

* $p \leq 0.05$; ** $p \leq 0.001$.

© 2020 by the authors. Licensee MDPI, Basel, Switzerland. This article is an open access article distributed under the terms and conditions of the Creative Commons Attribution (CC BY) license (http://creativecommons.org/licenses/by/4.0/).
